# Duodenal Aspirates for Small Intestine Bacterial Overgrowth: Yield, PPIs, and Outcomes after Treatment at a Tertiary Academic Medical Center

**DOI:** 10.1155/2015/971582

**Published:** 2015-01-28

**Authors:** Diana L. Franco, Molly B. Disbrow, Allon Kahn, Laura M. Koepke, Lucinda A. Harris, M. Edwyn Harrison, Michael D. Crowell, Francisco C. Ramirez

**Affiliations:** Division of Gastroenterology, Department of Medicine, Mayo Clinic Arizona, 13400 East Shea Boulevard, Scottsdale, AZ 85259, USA

## Abstract

Duodenal aspirates are not commonly collected, but they can be easily used in detection of small intestinal bacterial overgrowth (SIBO). Proton pump inhibitor (PPI) use has been proposed to contribute to the development of SIBO. We aimed to determine the yield of SIBO-positive cultures detected in duodenal aspirates, the relationship between SIBO and PPI use, and the clinical outcomes of patients identified by this method. In a retrospective study, we analyzed electronic medical records from 1263 consecutive patients undergoing upper endoscopy at a tertiary medical center. Aspirates were collected thought out the third and fourth portions of the duodenum, and cultures were considered to be positive for SIBO if they produced more than 100,000 cfu/mL. Culture analysis of duodenal aspirates identified SIBO in one-third of patients. A significantly higher percentage of patients with SIBO use PPIs than patients without SIBO, indicating a possible association. Similar proportions of patients with SIBO improved whether or not they received antibiotic treatment, calling into question the use of this expensive therapy for this disorder.

## 1. Introduction

Small intestinal bacterial overgrowth (SIBO) is a clinical entity that may be responsible for a constellation of symptoms that include bloating, abdominal distension, pain, and diarrhea. The diagnosis requires a positive culture defined as >100,000 colony forming units per mL (cfu/mL) in aspirates obtained from the small bowel [1]; however, some have used the less frequent and not as reliable cutoff of 10,000 cfu/mL [2]. Although breath tests have not been standardized, they may also serve as indirect evidence of SIBO when they are positive. SIBO has been associated with conditions affecting GI motility, such as gastroparesis and scleroderma, gastrointestinal surgery, small bowel diverticula, immunologic disorders, such as IgA deficiency and combined variable immunoglobulin deficiency, and conditions associated with decreased gastric acid secretion [2]. We sought to evaluate a cohort of patients undergoing EGD with duodenal aspirates to determine the diagnostic yield of cultures, clinical response to antibiotic treatment and the risk factors associated with SIBO.

Although there is controversy regarding the association between PPI therapy and SIBO, a recent meta-analysis has suggested a positive association only when the diagnosis of SIBO is based on aspiration cultures [3, 4]. We hypothesized that PPI use would be associated with higher rates of positive duodenal aspirate culture in our population.

## 2. Methods

The study was a retrospective review of patients undergoing outpatient EGD with duodenal aspirates at Mayo Clinic Arizona between January and December 2012. A list of these patients was retrieved from our endoscopy database. Indications for the procedure, demographic information, and endoscopic findings were recorded from the endoscopy reports. At endoscopy, duodenal aspirates were obtained via an aspiration catheter (Hobbs Medical Inc., Stafford Springs, CT, USA) and passed through the working channel of the upper endoscope (Olympus America Inc.) and its tip was positioned beyond the third or fourth part of the duodenum; in order to avoid any potential contamination, no suction of any esophagogastricduodenal secretions was performed prior to the positioning of the aspiration catheter in the duodenum The small bowel fluid was suctioned into a sterile container and at least 1 mL was obtained. The container was then immediately taken to the microbiology laboratory, where the aspirate was cultured for aerobic bacteria. The results were reported quantitatively, and growth of >100,000 cfu/mL was considered positive. No speciation was performed on the cultures. The medical records were then reviewed to document the results of duodenal aspirates and any subsequent clinical notes to document therapeutic intervention and its outcomes. The clinical outcomes were documented by either the referring physician or gastroenterology consultant or self-reported by the patient in telephonic communication with the healthcare team. Data regarding the current use of proton pump inhibitors and their doses at the time of EGD were recorded. Patients were divided into two groups according to documented clinical outcomes after primary treatment (i.e., antibiotics). The first group included patients whose symptoms completely resolved; the second group included patients whose symptoms did not resolve, resolved but recurred, or partially resolved. We obtained permission from our local IRB for the retrospective review, collection, and analysis of the data.

### 2.1. Statistical Analysis

Data were entered manually and statistically assessed using IBM SPSS (version 21.0; IBM SPSS, Chicago, IL). Student's *t*-tests were performed to evaluate means and differences in demographic variables.

Chi-square tests were used to compare proportional data. Logistic regression was performed to identify variables that might be predictive of clinical improvement. A *P* value less than 0.05 was considered statistically significant.

## 3. Results

**Figure 1:**
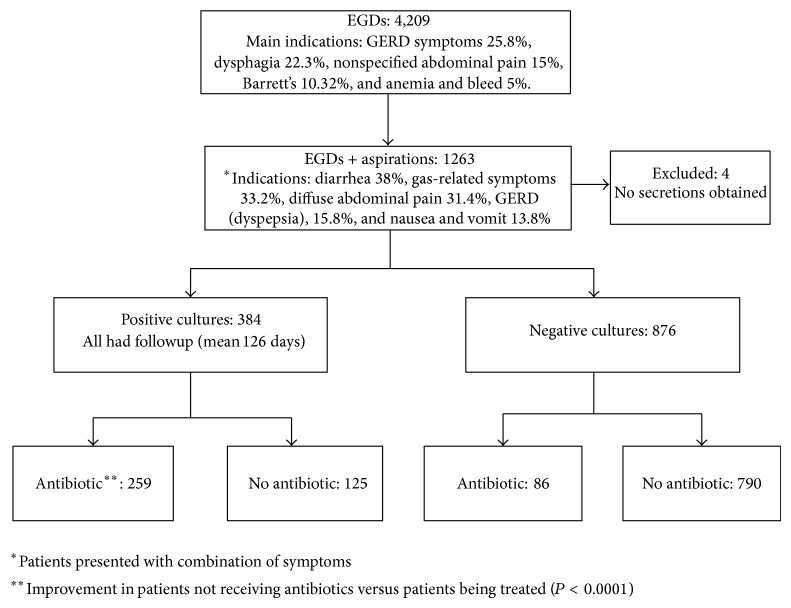


There were a total of 4,209 outpatient EGDs performed during the study period, of which 2,385 (56.7%) were performed in women. Duodenal aspirates were obtained in 1,263 (30%) patients undergoing these EGDs with a mean age of 52 years (16–93). Of these, 894 (71%) were in women and 369 (29%) in men (*P* < 0.0001). Four patients were excluded from the analysis as no cultures could be obtained because of the lack of duodenal secretions.  Figure 1 provides a summary of patient selection.

Table 1 shows the primary indications (alone or in combination) for EGDs with duodenal aspirates, with diarrhea being the most common (38%), followed by gas-related symptoms (33.2%) and diffuse abdominal pain (31.4%). Of the 1263 patients with positive cultures, 1055 patients (83%) presented with more than one symptom. Multivariant analysis was performed for the patients who had positive cultures and among all groups distributed by symptoms, diarrhea was the only variable statistically significant associated with clinical improvement.

The overall yield for positive (>100,000 cfu/mL) duodenal aspirates was 30.4%: 68.4% in women, and 31.5% in men (*P* < 0.001). PPIs were used in 466 (37%) patients; their use was similar in women (36.4%) and men (39.3%), and the duration of PPI use was between 0 and 180 months with a mean of 23.16 months and a median of 12 months. There was no correlation between diagnosis of SIBO and duration of PPI therapy. There were only 3 patients in whom a PPI was prescribed due to the presenting complaints. PPI use was significantly higher (52.6%) in patients with culture positive duodenal aspirates than those with culture negative aspirates (30.2%; *P* < 0.0001), suggesting a positive association between PPI and SIBO. There were no differences in the rate of high-dose PPI (defined as any standard prescription above the once a day dose) use between the culture positive and negative patients (Table 2). The use of PPI in culture positive and culture negative aspirates is described in Table 3.

Antibiotics were used in 67.4% of culture positive and 9.8% of culture negative patients. Of those with culture negative duodenal aspirates receiving antibiotics, quantitative culture was between 10,000 and 100,000 cfu/mL in 59.3% of patients. The antibiotic most commonly prescribed to patients with positive culture was Rifaximin (190 or 73.4%), followed by Ciprofloxacin (18 or 6.9%) and Metronidazole (13 or 5%). Similarly, patients with negative cultures were treated with Rifaximin (70 or 81.4%), Ciprofloxacin (4 or 4.7%), and Metronidazole (4 or 4.7%). The mean clinical follow-up period was 126 days (6–555 days). Although, overall, patients treated with antibiotics showed greater clinical improvement than those not receiving antibiotics (53.1% versus 24.6%; *P* < 0.0001), in the cohort of patients with positive cultures, clinical improvement did not differ significantly whether antibiotic was given or not; there was clinical improvement in 53% in patients treated with antibiotic versus 46.5% in patients not treated with antibiotic.

We found that, in patients with positive cultures, clinical improvement, irrespective of antibiotic use, correlated with diarrhea on presentation.

## 4. Discussion

SIBO has been traditionally defined according to the number of culturable bacteria in duodenal or jejunal aspirates. Most authors consider culture of small bowel aspirates the gold standard method and a quantitative bacterial culture of ≥10^5^ cfu/mL as a positive diagnosis of SIBO [2, 5]. The quantitative diagnosis is based on data that normal colony counts in the proximal small intestine are in the order of 10^2^ and are mainly composed of lactobacilli, enterococci, gram-positive aerobes, and facultative anaerobes [6, 7]. The sensitivity and specificity of small bowel aspirate cultures for the diagnosis of SIBO approach 100% [8, 9]. However, because the test is considered invasive and requires the performance of an upper endoscopy, breath testing with various substrates (glucose, lactulose, D-xylose, and C-glycocholate) has been developed as an indirect diagnostic method. Unfortunately, these indirect methods are not standardized and their overall sensitivities and specificities have been low, ranging from 6 to 33% and 44% to 100%, respectively [5, 8]. Potential contributors to the low sensitivity include increased conversion of hydrogen to methane by certain gut microbes, hyperventilation from recent exercise or pulmonary disease, increased oral bacteria, and low anaerobic bacterial load in the colon [10, 11]. A recent meta-analysis [8] has nicely outlined the different sensitivities and specificities of available tests for the diagnosis of SIBO. The advent of techniques using nucleic acid-based strategies and metagenomics to define the gut ecosystem may change the definition of the diagnosis of SIBO in the future [12].

We found that SIBO was not only clinically suspected but confirmed more often in women than men and that the condition was highly prevalent in the cohort of patients undergoing duodenal aspirates (30%) at our tertiary referral center. In a recent cohort study, a total of 675 patients had available aspirate results and 8% of aspirates were positive for SIBO. Older age, steatorrhea, and narcotic use were associated with SIBO (*P* < 0.05). PPI use was not associated with SIBO in that study but was associated with bacterial growth not meeting the commonly used (>100,000 cfu/mL) criteria for SIBO [13]. SIBO may be clinically asymptomatic or can include nonspecific symptoms such as bloating, flatulence, abdominal discomfort, diarrhea, and abdominal pain. To our knowledge, there is no correlation between amount of bacteria and severity of symptoms. In our study, symptoms varied and included abdominal pain, diarrhea, bloating, nausea, dysphagia, emesis, and dyspepsia. The most prevalent symptoms in our patients with SIBO were diarrhea and bloating (32%) and the majority of patients (83%) had more than one symptom at the time of endoscopy.

Conditions that disrupt defense mechanisms against bacterial overgrowth [14] (gastric acid secretion, intestinal motility, intact ileocecal valve, pancreatic secretions, and intestinal immunoglobulins) are known to predispose to SIBO.

A study of a specific IBS-refractory group of patients found that increased counts of bacteria from jejunal aspirates [15] were more common in these patients, but no causative role could be identified between the type of altered motility in these IBS patients and SIBO. In contrast to that study, our patients underwent duodenal aspirates based on symptoms and not on diagnoses (i.e., IBS, celiac disease, etc.) and duodenal aspirates rather than jejunal aspirates were used for diagnosing SIBO. This is also important as IBS patients could profit from antibiotic treatment because of dysbiosis within the colon not necessary associated with SIBO.

Similarly, PPI therapy, which is commonly used in clinical practice, has been suggested as a risk factor for the development of SIBO, although the literature has been controversial and contradictory. This in part is due to the different methods used to diagnose SIBO and the overall lack of standardization. Some studies have found no correlation between the use of PPIs and SIBO as measured by indirect breath tests [3] whereas others, using small bowel aspirates, have shown a higher association of SIBO with PPIs when compared to the least potent gastric antisecretory therapy [16]. As stated earlier, there are several diagnostic methods that have been used to diagnose SIBO. In the study by Gabbard et al. [4], where lactulose breath test was used to diagnose SIBO, there was a link found between PPI use and the diagnosis of SIBO; in contrast to that study, we utilized duodenal aspirates. A recent meta-analysis [6] found that there is a positive association between PPI use and SIBO only when duodenal or jejunal aspirate culture, and not breath testing, is performed. Our results are in agreement with these findings. It is worth to mention that data regarding PPI use were recorded only at the time of endoscopy and continuation or discontinuation of PPI treatment during the follow-up period could contribute to the results.

Once the diagnosis of SIBO is established, the treatment is aimed at reducing or eliminating the bacterial overgrowth with antibiotic therapy, hopefully leading to the resolution of symptoms. There is no consensus regarding the antibiotic of choice for SIBO. Historically, treatment regimens have included cephalosporins, penicillins, tetracyclines, and fluoroquinolones with mixed results [13]. In studies using breath tests, norfloxacin and amoxicillin clavulanate [17] have been reported to be effective in relieving symptoms. Studies comparing a 10-day course of rifaximin to placebo have not shown a significant difference in symptom relief or posttreatment breath testing [18]. Interestingly, although patients receiving antibiotics improved their symptoms overall, we did not find significant differences in symptom resolution among culture positive patients treated with and without antibiotics. This raises the question as to whether the choice of an expensive antibiotic therapy (e.g., rifaximin) is superior to no treatment at all or other less expensive strategies and that perhaps there is a subgroup of patients (those presenting with diarrhea as their predominant symptom) who would benefit the most from the treatment.

Limitations of our study included its retrospective nature, a study population that included a selected group of patients referred to a single tertiary referral center, the subjective assessment of clinical improvement or lack thereof after therapeutic intervention, and the lack of randomization to treatment options (antibiotics versus no antibiotic).

In conclusion, duodenal aspirates were performed in 30% of our outpatient EGD practice and the yield for positive cultures was 30%. The use of PPIs was significantly higher in the patients with positive cultures than in patients with negative cultures, suggesting a possible association. In patients with positive cultures, clinical improvement was similar irrespective of antibiotic treatment; this opens a debate to evaluate the pertinence of antibiotic treatment and even duodenal aspirates themselves as a diagnostic modality. We believe that further studies should aim to characterize the population that may derive the most benefit and evaluate the associated costs of diagnostic and therapeutic strategies.

## Figures and Tables

**Table 1:** Indications for EGD with duodenal aspirates.

Indication	Frequency (%)	Frequency of (+) Cx per indication	Frequency of improvement per indication
Diarrhea	480 (38%)	126 (26.2%)	60 (47.6%)
Gas-related symptoms (gas, bloating, distention, eructation, and flatus)	419 (33.2%)	140 (33.4%)	60 (42.8%)
Diffuse/upper abdominal pain^*^	397 (31.4%)	116 (29.2%)	39 (33.6%)
Dyspepsia/GERD^*^	199 (15.8%)	69 (34.6%)	3 (4.3%)
Nausea/vomiting	174 (13.8%)	62 (35.6%)	12 (19.3%)

^*^Part of symptoms combination except in 25% of patient who had persistent pain and 40% that had persistent GERD symptoms.

**Table 2:** Standard versus high PPI dose in culture positive and culture negative patients.

PPI	Culture positive	Culture negative
High dose	53/192 (27.6%)	53/217 (24.4%)
Standard dose	137/192 (71.4%)	152/217 (70%)

**Table 3:** PPI use in culture positive and culture negative aspirates.

	Culture positive	Culture negative
	(52.6%)^*^	(30.2%)^*^
Omeprazole	88/192 (45.8%)	81/217 (37.3%)
Esomeprazole	38/192 (19.8%)	39/217 (18%)
Pantoprazole	32/192 (16.7%)	44/217 (20.3%)
Lansoprazole	15/192 (7.8%)	32/217 (14.7%)
Rabeprazole	12/192 (6.3%)	7/217 (3.2%)
Dexlansoprazole	7/192 (3.6%)	14/217 (6.5%)

^*^PPI use in patients with positive versus negative cultures (*P* < 0.0001).
